# Comparative transcriptomes analysis of the wing disc between two silkworm strains with different size of wings

**DOI:** 10.1371/journal.pone.0179560

**Published:** 2017-06-15

**Authors:** Jing Zhang, Danso Blessing, Chenyu Wu, Na Liu, Juan Li, Sheng Qin, Muwang Li

**Affiliations:** 1 School of Biotechnology, Jiangsu University of Science and Technology, Zhenjiang, Jiangsu, China; 2 The Sericultural Research Institute, Chinese Academy of Agricultural Science, Zhenjiang, Jiangsu, China; Institute of Plant Physiology and Ecology Shanghai Institutes for Biological Sciences, CHINA

## Abstract

Wings of *Bombyx mori* (*B*. *mori*) develop from the primordium, and different *B*. *mori* strains have different wing types. In order to identify the key factors influencing *B*. *mori* wing development, we chose strains P50 and U11, which are typical for normal wing and minute wing phenotypes, respectively. We dissected the wing disc on the 1^st^-day of wandering stage (P50D1 and U11D1), 2^nd^-day of wandering stage (P50D2 and U11D2), and 3^rd^-day of wandering stage (P50D3 and U11D3). Subsequently, RNA-sequencing (RNA-Seq) was performed on both strains in order to construct their gene expression profiles. P50 exhibited 628 genes differentially expressed to U11, 324 up-regulated genes, and 304 down-regulated genes. Five enriched gene ontology (GO) terms were identified by GO enrichment analysis based on these differentially expressed genes (DEGs). KEGG enrichment analysis results showed that the DEGs were enriched in five pathways; of these, we identified three pathways related to the development of wings. The three pathways include amino sugar and nucleotide sugar metabolism pathway, proteasome signaling pathway, and the Hippo signaling pathway. The representative genes in the enrichment pathways were further verified by quantitative real-time reverse transcription polymerase chain reaction (qRT-PCR). The RNA-Seq and qRT-PCR results were largely consistent with each other. Our results also revealed that the significantly different genes obtained in our study might be involved in the development of the size of *B*. *mori* wings. In addition, several KEGG enriched pathways might be involved in the regulation of the pathways of wing formation. These results provide a basis for further research of wing development in *B*. *mori*.

## Introduction

*Bombyx mori* (*B*. *mori*) was domesticated from the wild silkworm (*B*. *mandarina*) It is classified as a Lepidopteran insect, making it an ideal species to study the biochemistry, molecular genetics, and genomics of Lepidoptera. It has been intensively studied for the past several decades owing to its economic and academic value [1].

The wing is one of the important organs of insects, having many functions such as flight, protection, communication, orientation, and courtship [2]. Larval primordium of holometabolous insects gives rise to the wing of the adult during development. During this process, a drastic change occurs in the wing primordium. In the larval stage, the wing discs proliferate gradually, and then, during pupal ecdysis, extensive proliferation and morphological changes occur to enable the wing to evaginate from inside the body. There are several hormones involved in this biological regulation process, such as 20-hydroxyecdysone (20E) and juvenile hormone (JH). 20E and JH are two major hormones in insects which regulate different biological processes, including growth, molting, and reproduction [3, 4].

A comprehensive profile of *B*. *mori* gene expression during wing disc metamorphosis has been revealed by RNA-sequencing (RNA-Seq) [5]. RNA-Seq is based on deep sequencing technology, which is a powerful and cost-efficient tool for transcriptome analysis. Transcriptome analysis can result in the complete set of RNA transcripts produced by the genome at one time, and an understanding of the transcriptome is essential for interpreting the functional elements of the genome [6]. In recent years, RNA-Seq has been widely used. Li used RNA-Seq technology to obtain thousands of different genes in silk glands between two silkworm strains, for example, and provided a perspective for understanding the molecular mechanisms determining silk yield [7]. Connahs and Ou used RNA-Seq to compile a comprehensive list of differentially expressed transcripts during wing development in *Vanessa cardui* and *B*. *mori* [5, 8].

In eukaryotic cells, the degradation of many proteins involves their covalent modification by conjugation with ubiquitin. Ubiquitinated proteins can be rapidly degraded by a large, multi-subunit complex called the 26S proteasome [9]. Previous research has found that dominant-negative mutations in the b2 and b6 proteasome subunit genes affect alternative cell fate decisions in the *Drosophila* sense organ lineage [10]. This study revealed that the proteasome is associated with organ cell fate. There are few reports, however, on the role of the proteasome in wing development between different strains of silkworm.

Initially discovered in *Drosophila*, the Hippo (Hpo) pathway has been recognized as a conserved signaling pathway that controls organ size during development. It does this by restricting cell growth and proliferation, and promoting apoptosis [11]. A lot of research suggests that the Hippo signaling pathway is gaining recognition as an important player in both organ size control and tumorigenesis; physiological and pathological processes that share common cellular signaling mechanisms [12, 13]. Limited analyses were performed on the signal pathway for organ size control in *B*. *mori*. In our study, we found that the expression of genes have significant differences in the Hippo pathway between P50 and U11.

Previous studies have focused mainly on the wing disc transcriptome during metamorphosis [5], but there appears to be no study on the wing disc transcriptome between two strains which have different sized wings. In the present study, RNA-Seq was used to attain a comprehensive view of gene expression in the *B*. *mori* wing disc between P50 and U11: common strains with normal wing and minute wing, respectively [6]. Our results would provide background information for further research on wing development in *B*. *mori*.

## Materials and methods

### Experimental animal and RNA isolation

P50 and U11 strains, which have different wing sizes, were chosen from the silkworm resource library. The larvae were reared on live mulberries under constant 14-h light/10-h dark photoperiod, at 25 ± 1°C, and 75% ± 3% relative humidity.

Intact wing discs were dissected and immediately frozen in liquid nitrogen for RNA-Seq. During the dissection, the wing discs were dissected completely, then were washed with the saline in order to remove HPOs.

### RNA extraction and library preparation

*B*. *mori* wing discs were clearly dissected from 1^st^-day of wandering stage, 2^nd^-day of wandering stage, and 3^rd^-day of wandering stage, from both P50 and U11 strains. Then, the total RNA was extracted from primordium wings using TRIzol reagent (Invitrogen, USA), following the manufacturer's protocol. Equal amounts of RNA from three days samples were mixed for P50 and U11 respectively.

A total of 3 μg RNA per sample was used as input material for the RNA sample preparations. Sequencing libraries were generated using NEBNext^®^ Ultra^™^ RNA Library Prep Kit for Illumina^®^ (NEB, USA), following the manufacturer’s recommendations, and index codes were added to attribute sequences to each sample.

### RNA sequencing (RNA-Seq) and quality control

The clustering of the index-coded samples was performed on a cBot Cluster Generation System, using TruSeq PE Cluster Kit v3-cBot-HS (Illumina), according to the manufacturer’s instructions. After cluster generation, the library preparations were sequenced on an Illumina Hiseq 2500 platform and 150bp paired-end reads were generated. Raw data (raw reads) of fastq format were firstly processed through in-house perl scripts. In this step, clean data (clean reads) were obtained by removing reads containing adapters, poly-N, and low quality reads from the raw data. All the downstream analyses were based on high quality, clean data. Reference genome and gene model annotation files were downloaded from Silkworm Genome Database: http://silkworm.genomics.org.cn/ and KAIKObase: http://sgp.dna.affrc.go.jp/KAIKObase/ [14, 15].

### Differential expression analysis of genes

HTSeq v0.6.1 was used to count the number of reads mapped to each gene. The FPKM (the expected number of Fragments Per Kilobase of transcript sequence per Millions base pairs sequenced) of each gene was then calculated based on the length of the gene and read count mapped to that gene. FPKM considers the effect of sequencing depth and gene length for the read counts, and is currently the most commonly used method for estimating gene expression levels [16].

Differential expression analysis of two conditions was performed using the DEGSeq R package (1.20.0) [17]. The *P* values were adjusted using the Benjamini & Hochberg method. Corrected *P*-value of 0.005 and log2(Fold change) of 1 were set as the threshold for significantly differential expression [18].

### GO and KEGG enrichment analysis of differentially expressed genes

Gene Ontology (GO) enrichment analysis of differentially expressed genes was implemented by the GOseq R package [19], in which gene length bias was corrected. GO terms with corrected *P*-value less than 0.05 were considered significantly enriched by differentially expressed genes [20].

KEGG is a database resource for understanding high-level functions and utilities of the biological system, such as the cell, the organism and the ecosystem, especially large-scale molecular datasets generated by genome sequencing and other high-throughput experimental technologies (http://www.genome.jp/kegg/) [21]. We used KOBAS software to test the statistical enrichment of differentially expression genes in KEGG pathways [22, 23].

### Validation by quantitative real-time reverse transcription polymerase chain reaction (qRT-PCR)

To further validate the RNA-Seq results, wing discs from three biological samples of each strain were dissected from 1^st^-day of wandering stage, 2^nd^-day of wandering stage, and 3^rd^-day of wandering stage (P50D1, P50D2, P50D3, U11D1, U11D2, U11D3). RNAiso Plus (TaKaRa Dalian, China) was used to isolate total RNA, according to the manufacturer’s instructions. Gel electrophoresis and ultraviolet spectrophotometry were used to determine the integrity and purity of the RNA. One μg of total RNA from each sample was used to synthesize cDNA, using a PrimeScriptTM RT reagent Kit with gDNA Eraser (Perfect Real Time, TaKaRa), followed by storage at -20°C. Real-time quantitative PCR was carried out in a reaction volume of 20 μL, containing 2 μL of template, 10 μL of 2× SYBR Premix EX Taq (TaKaRa), 0.4 μL of 50× ROX Reference Dye (TaKaRa), and 0.4 μL of specific primers (10 μM). The PCR amplification efficiency (E) and R^2^ of each primer pair was calculated from the slope of a standard curve, which was conducted according to MIQE (Minimum information for publication of quantitative real-time PCR experiments) guidelines [24]. The qRT-PCR primer sequences were designed based on the consensus sequence of each alignment, and their efficiencies are provided in S1 Table. qRT-PCR was performed with an ABI7300 real-time PCR system, using the following conditions: 95°C for 5 min, followed by 40 cycles of 95°C for 5 s, 60°C for 31 s and dissociation.

The mRNA quantity of each gene was calculated with the 2-ΔΔCt method [25], and normalized to the abundance of the house-keeping gene, BmGAPDH (Accession No. XM_012690444) [26]. The relative mRNA levels of each gene are presented as fold-change relative to the expression level of BmGAPDH.

The expression levels of each gene in the two respective strains were compared using a Student's *t*-test. Differences in gene expression between the two strains were considered significant at *P* ≤ 0.05.

## Results

### Overview of transcriptome sequencing data

**Fig 1:**
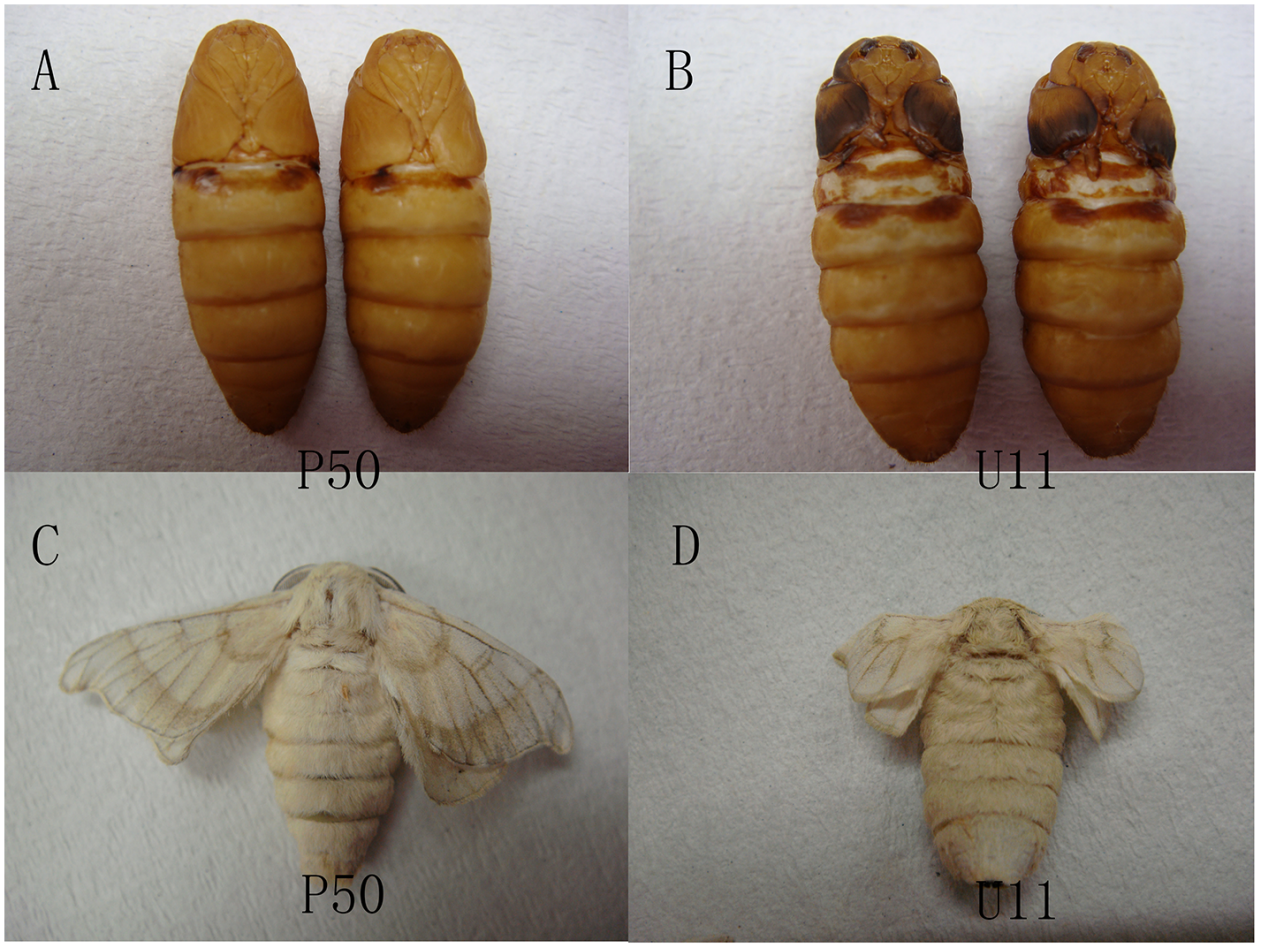
The wings of pupa and adults of P50 and U11. (A) The pupa of P50 male (left) and female (right). (B) The pupa of U11 male (left) and male (right). (C) The wings of P50. (D) The wings of U11.

**Fig 2:**
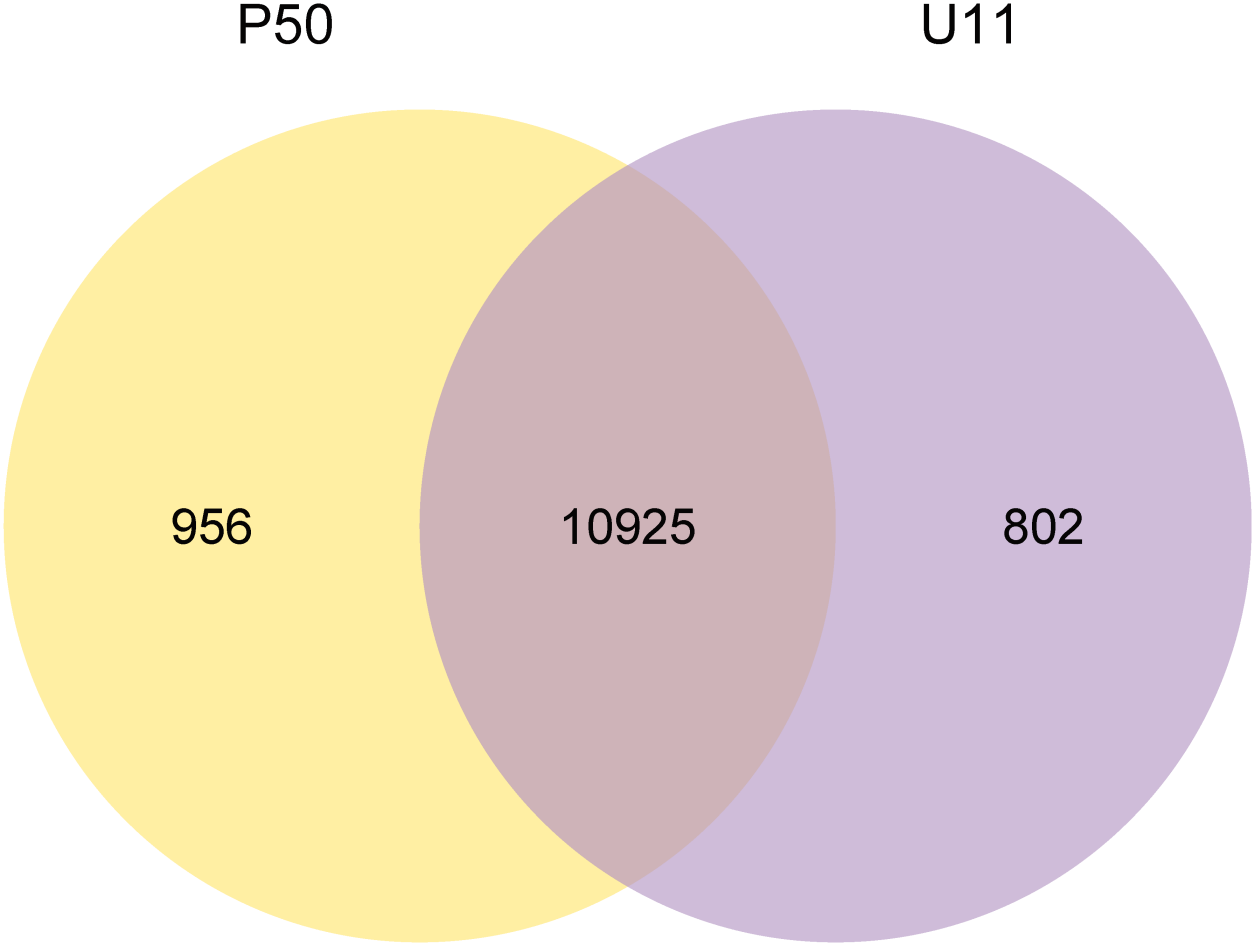
Statistics of transcript numbers and distribution at P50 and U11.

We choose P50 and U11 strains, which have different wing sizes (Fig 1). Total RNAs were extracted from wing discs, and cDNA libraries were then constructed and sequenced using an Illumina HiSeq2500 platform. 56,892,518 and 50,680,302 raw reads were generated from cDNA libraries. The quality of RNA-Seq is listed in S2 Table-1. In total, 54,744,438 and 48,631,418 clean reads were obtained from the P50 and U11, respectively. A total of 46,386,612 (84.73%) clean reads were mapped to 11,881 genes in P50, and 32,128,459 (66.07%) clean reads were mapped to 11,727 genes in U11 (S2 Table-2). There were 10,925 commonly expressed genes between the P50 and U11 strains. These results show that most genes are commonly expressed in P50 and U11, and the differentially expressed genes might be important for the wing size (Fig 2).

### Differentially expressed genes (DEGs) and gene ontology (GO) enrichment analysis

**Fig 3:**
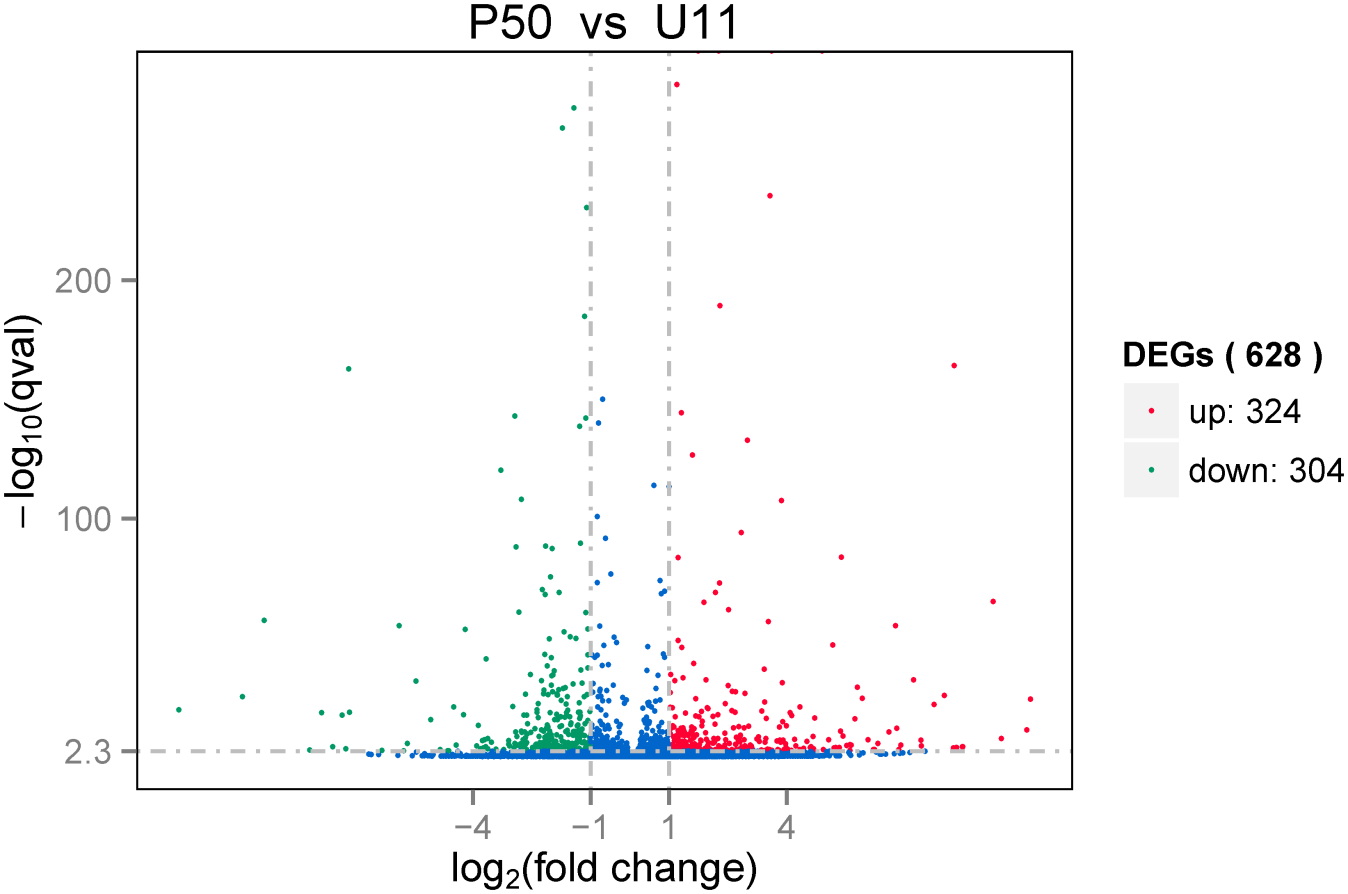
Volcano plot of DEGs. The red points indicate genes up-regulated in P50, green points indicate the genes down-regulated in P50, and blue points indicate genes for which the log2 fold change was less than 1 (and are therefore not differentially expressed between P50 and U11).

The gene expression levels were compared between the two strains using DEGSeq. Based on the thresholds for screening DEGs, 628 genes were identified as DEGs in P50 compared to U11; of these, 324 were up-regulated, and 304 were down-regulated in P50 (Fig 3 and S3 Table).

Differentially expressed genes between the two strains may affect the development of wings. Therefore, GO enrichment analysis of differentially expressed genes may reveal the reason for the different sized wings. Gene Ontology (GO) enrichment analysis of differentially expressed genes was implemented by the GOseq R package [19]. The results showed that the structural constituent of the cuticle, structural molecule activity, extracellular region, chitin binding, and aminoglycan metabolic processes were the most abundant GO function items when compared P50 to U11 (Table 1).

**Table 1:** Gene ontology enrichment of the DEGs between P50 and U11.

GO item	Cluster frequency	Genome frequency of use	*P* value	Corrected *P* value
structural constituent of cuticle	28 /408 genes	144 /9990 genes	2.13E-13	8.64E-10
structural molecule activity	47 /408 genes	626 /9990 genes	4.09E-05	8.31E-2
extracellular region	43 / 408 genes	543 /9990 genes	1.63E-4	2.20E-1
chitin binding	9 / 408 genes	50 / 9990 genes	3.31E-4	3.36E-1
aminoglycan metabolic process	11/ 408 genes	78 /9990 genes	5.98E-4	4.86E-1

Specifically, in biological process ontology, no dominant GO items were found in the genes up-regulated in the P50 strain, while several dominant GO items were found in the down-regulated genes. These dominant GO items include aminoglycan metabolic process, chitin metabolic process, glucosamine-containing compound, amino sugar metabolic process, defense response, regulation of localization, lipid transport and lipid localization. Compared with U11, the dominant GO items of the significantly up-regulated transcripts over down-regulated transcripts for P50 are purine nucleotide metabolic process.

Compared with U11, there are all significantly dominant GO items of the down-regulated transcripts over the up-regulated transcripts for P50 in cellular component ontology. However, no dominant GO items of the up-regulated transcripts were found, except in the extracellular region.

**Fig 4:**
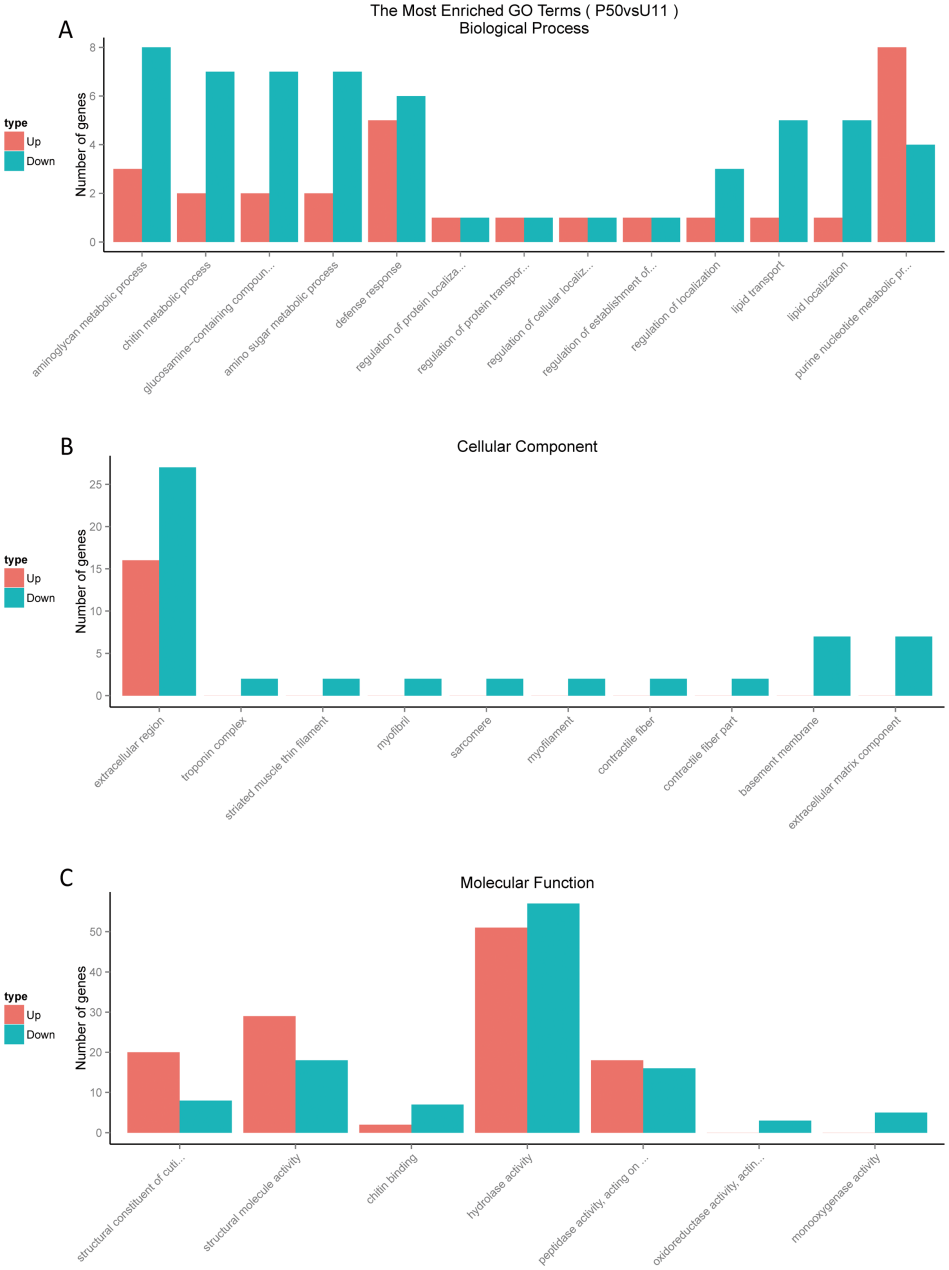
GO classification of the differentially expressed genes. (A) These genes were annotated as biological processes. (B) These genes were annotated as cellular components. (C) These genes were annotated as molecular functions. The up-regulated genes are marked with a pink bar and down regulated genes are marked with a blue bar.

For molecular function ontology for P50 vs. U11, the dominant GO items of the up-regulated transcripts included structural constituent of cuticle, structural molecule activity, and peptidase activity, acting on L-amino acid peptides. Specifically, the structural constituent of cuticle and structural molecule activity were significantly enriched (*p* value < 0.05). The dominant GO items of the down-regulated genes include chitin binding, hydrolase activity, oxidoreductase activity, and monooxygenase activity (Fig 4 and S4 Table).

### KEGG enrichment analysis of the differentially expressed genes

KOBAS software was used for KEGG enrichment analysis. The DEGs between the two silkworm strains were significantly enriched in five pathways (Table 2 and S5 Table). Of these, three pathways were identified that were related to the development of wings, including amino and nucleotide sugar metabolism, the proteasome pathway, and the fly Hippo signaling pathway. The proteasome and Hippo signaling pathways were significantly up-regulated in the P50 strain, while the others were significantly down-regulated.

**Table 2:** KEGG enrichment of the DEGs between P50 and U11.

KEGG pathway	Sample number	Background number	*P* value	UniGenes	KO
Tyrosine metabolism	4	16	1.27E-2	BGIBMGA002958 BGIBMGA007438 BGIBMGA000563 BGIBMGA003199	bmor:101745777 bmor:693082 bmor:100270767 bmor:692675
Proteasome	6	38	1.59E-2	BGIBMGA003335 BGIBMGA011237 BGIBMGA009562 BGIBMGA005014 BGIBMGA010794 BGIBMGA013898	bmor:101745852 bmor:693113 bmor:732876 bmor:101745357 bmor:733024 bmor:692836
Amino sugar and nucleotide sugar metabolism	7	52	1.96E-2	BGIBMGA007517 BGIBMGA007425 BGIBMGA014523 BGIBMGA008064 BGIBMGA001609 BGIBMGA004859 BGIBMGA006874	bmor:101743496 bmor:101743212 bmor:101739517 bmor:101744393 bmor:101746744 bmor:101743965 bmor:692403
Hippo signaling pathway—fly	7	55	2.51E-2	BGIBMGA008411 BGIBMGA002237 BGIBMGA003681 Novel01133 BGIBMGA013945 BGIBMGA003592 BGIBMGA003591	bmor:101742023 bmor:101741612 bmor:100144579 bmor:101746395 bmor:100145913 bmor:692948 bmor:692948
Phenylalanine metabolism	3	12	3.06E-2	BGIBMGA002958 BGIBMGA003866 BGIBMGA003199	bmor:101745777 bmor:101742825 bmor:692675

### Validation of DEGs by qRT-PCR

To validate the RNA-Seq data, qRT-PCR was performed on significantly differentially expressed genes selected from the enrichment pathways. The gene sequences were obtained from the silkworm genome sequence [1]. The qRT-PCR expression results were similar to the results obtained from the Illumina sequencing data.

**Fig 5:**
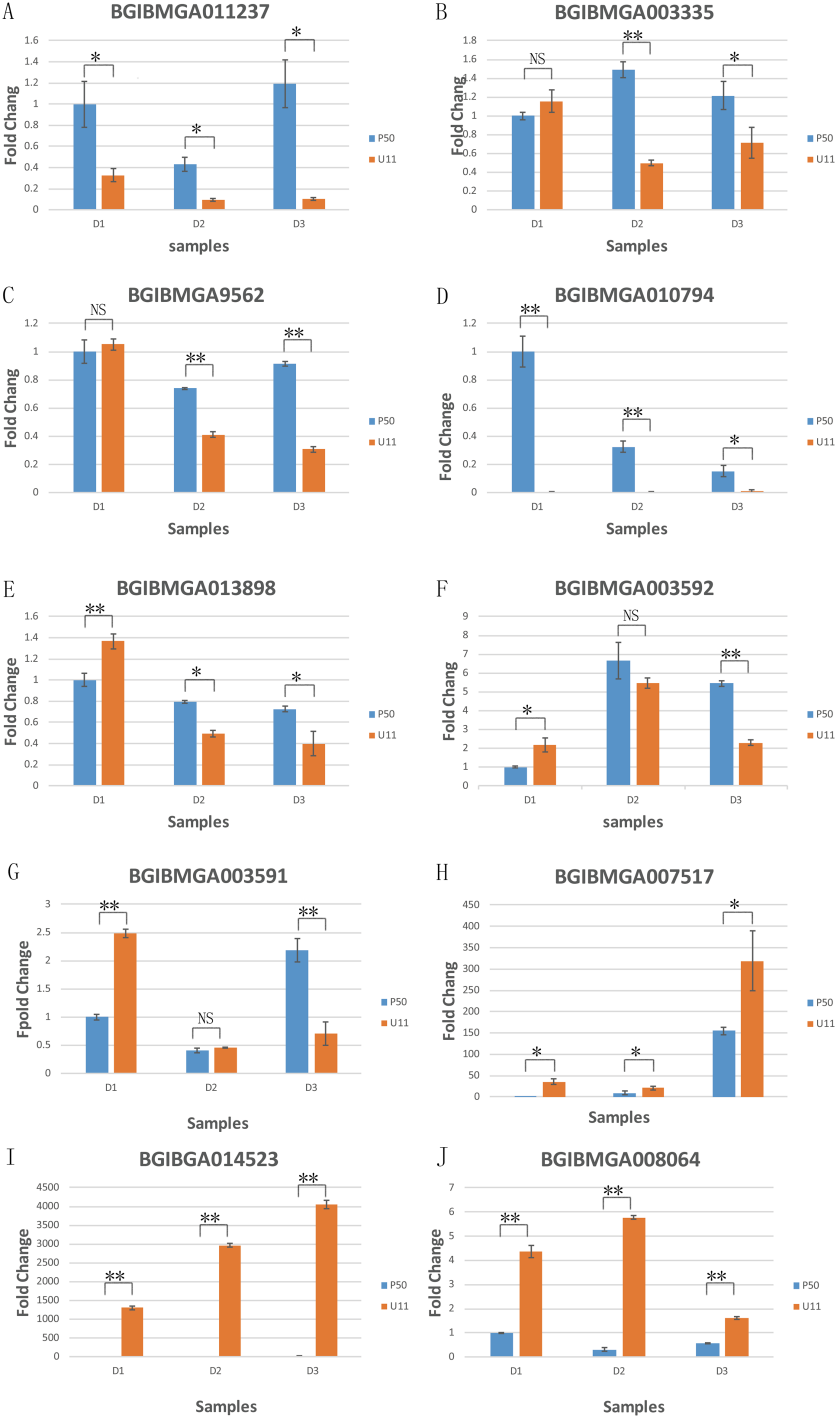
Quantitative real-time PCR validation for genes. Blue bars indicate gene expression in P50 and orange bars indicate gene expression in U11. ‘*’ indicates significant difference (0.01<*P*-value ≤0.05); ‘**’ indicates the most significant difference (*P*-value<0.01). A-E are related to proteasome enriched genes, F and G are involved in the Hippo signaling pathway, H-J are related to amino sugar and nucleotide sugar metabolism. **D1:** 1^st^-day of wandering stage; **D2;** 2^nd^-day of wandering stage; **D3:** 3^rd^-day of wandering stage.

Based on the qRT-PCR results, we found that the following genes were differently expressed in the P50 and U11. Genes associated with proteasome and the Hippo signaling pathway, including *BGIBMGA003335*, *BGIBMGA0011237*, *BGIBMGA009562*, *BGIBMGA010794*, *BGIBMGA13898*, *BGIBMGA003592*, and *BGIBMGA003591*, showed higher expression in the wing disc of P50. Genes associated with amino sugar and nucleotide sugar metabolism, including *BGIBMGA007517*, *BGIBMGA0014523*, and *BGIBMGA008064*, showed lower expression in the wing disc of P50 (Fig 5). The *p*-values was showed in S6 Table.

### The proteasome and Hippo signaling pathway were more active in P50

Proteasomes are highly conserved proteinase complexes that have been found in archaebacteria and all examined eukaryotes [27]. Biochemical studies have shown that proteasomes exhibit multiple endopeptidase activities, and that they can exist in active and latent forms [28]. The Hippo (Hpo) pathway has been recognized as a conserved signaling pathway that controls organ size during development by restricting cell growth and proliferation, and by promoting apoptosis [11]. It is reasonable, therefore, to hypothesize that enrichment of genes in these two pathways is associated with organ size. To investigate activity of the proteasome and Hippo signaling pathways, the expression patterns of the genes that are involved in the two pathways were identified and analyzed.

The results revealed that most of the genes that are associated with the proteasomes pathway, such as *BGIBMGA003335*, *BGIBMGA011237*, *BGIBMGA009562*, *BGIBMGA010794*, and *BGIBMGA013898*, and most of the genes that are associated with the Hippo pathway, such as *BGIBMGA003591* and *BGIBMGA003592*, were all significantly up-regulated in P50 (Fig 5A–5G). This implied that these two pathways are actively involved in wing disc size.

### The amino sugar and nucleotide sugar metabolism were active in U11

It has already been proven that sugar metabolism has an important influence on organ size, so it is reasonable to hypothesize that enrichment of genes in this pathway is associated with organ size. To investigate the activity of the amino sugar and nucleotide sugar metabolism pathway, the expression patterns of the genes that are involved in these two pathways were identified and analyzed.

The results revealed that most of the genes that are associated with the amino sugar and nucleotide sugar metabolism, such as *BGIBMGA007517*, *BGIBMGA0014523*, and *BGIBMGA008064*, were all significantly up-regulated in U11 (Fig 5H–5J). This implied that the activity of the amino sugar and nucleotide sugar metabolism is involved in the wing disc size.

## Discussion

The wing is one of the most important organs found in insects. Different species have different size of wings, and wing size is controlled by many factors. In this research, P50 originated from Chinese Guangdong province, named Dazao in China, it is a wild type for the wing. U11 is a recessive mutant was found as a spontaneous occurrence in a hybrid stock of d 30. (Fig 1). The marks are different between these two strains, P50 is normal mark (+p), while U11 is plain (p), there are no other morphological differences between these two strains. In *Drosophila*, the basis of an overgrowth phenotype is driven by the *Drosophila* protein, Crumbs (Crb), which nucleates an apical membrane complex that functionally interacts with the Par6/Par3/aPKC and Scrib/Dlg/Lgl apicobasal polarity complexes. Studies have shown that treatment with a proteasome inhibitor can retard the effect of Crb^i^, resulting in smaller organs [29]. This is consistent with our transcriptome sequencing data, which showed that six genes were enriched and up-regulated in the proteasome pathway of the *B*. *mori* P50 strain. Our qRT-PCR results showed that the expression level of *BGIBMGA003335*, *BGIBMGA011237*, *BGIBMGA009562*, *BGIBMGA010794*, and *BGIBMGA013898* was up to two to six times higher in the P50 strain than in U11, in 2nd day and 3rd day (Fig 5A–5E). In addition, we found that *BGIBMGA003335* is associated with the formation of the b6 proteasome. A study also showed that Crb regulates Salvador/Warts/Hippo Signaling in *Drosophila* via the FERM-Domain Protein Expanded [29]. It is reasonable to believe, therefore, that proteasome is associated with the size of silk worm wings.

The Hippo signaling pathway is associated with organ size control and tumorigenesis [30]. In *Drosophila*, the signaling pathways upstream of the membrane protein receptors sense the extracellular growth-inhibiting signal, phosphorylation then cascades through a series of kinase complexes, ultimately phosphorylating the downstream effector factor, Yki [31]. The phosphorylated Yki interacts with cytoskeletal proteins, allowing it to be trapped in the cytoplasm and not to enter the nucleus to effect transcriptional activation [12]. Previous research has shown that Yki is a transcriptional coactivator; the unphosphorylated form activates the expression of transcriptional targets that promote cell growth, cell proliferation, and prevent apoptosis [32]. Consequently, up-regulation of the Hippo signaling pathway inhibits Yki phosphorylation and regulates organ size (http://www.genome.jp/kegg-bin/show_pathway?bmor04391+100144579). Previous research has shown that Yki is a transcriptional coactivator, unphosphorylated form to activate expression of transcriptional targets that promote cell growth, cell proliferation, and prevent apoptosis [32].

According to our RNA-seq results, 5 genes were enriched in the Hippo signaling pathway, such as *BGIBMGA003592* and *BGIBMGA003591*, all of which were mostly up-regulated in P50. In *Drosophila*, two of these genes are associated with Lix1 protein, which is involved in the Hippo signal pathway. qRT-PCR was performed for the two significantly different genes, and the results show that, overall, from D1 to D3, their expression levels increased in P50. The results also showed that, from D1 to D3, the expression levels decreased in U11. In general, the expression levels of the Hippo signaling pathway genes were higher in P50 (Fig 5F and 5G).

We speculate that in the development of U11 wing primordium, the Hippo signaling pathway down-regulation leads to the Yki phosphorylation. This means it cannot enter the nucleus to effect transcriptional activation, and thus leads to minute wings.

In our study, we also found that genes associated with amino sugar and nucleotide sugar metabolism are significantly down-regulated in the P50 strain. We selected three significantly differentially expressed genes from the pathway that were associated with organ size development: *BGIBMGA007517*, *BGIBMGA014523*, and *BGIBMGA008064*. qRT-PCR was then performed for these three genes. The results showed that all these genes are significantly down-regulated from D1 to D3 in the P50 strain compared with the U11 strain. Therefore, it is possible that these genes are involved in *B*. *mori* wing disc metamorphosis.

Previous studies have shown that these genes are involved in sugar metabolism. Moreover, identifying that DEGs between P50 and U11 were enriched in amino sugar and nucleotide sugar metabolism in this study suggest a relationship between sugar metabolism and organ size. We identified the *BGIBMGA007517* gene, which functions as glutamine-fructose-6-phosphate aminotransferase, the *BGIBMGA014523* gene, which functions as *B*. *mori* N-acetylneuraminate lyase-like, and the BGIBMGA008064 gene, which function as chitooligosaccharidolytic beta-N-acetylglucosaminidase-like.

Previous research has shown that the enzyme activity is necessary for the induction of TGF-β1 and fibronectin expression in mesangial cells. Increased flux through the hexosamine biosynthetic pathway with the rate-limiting enzyme, glutamine-fructose-6-phosphate aminotransferase (GFAT), has been linked to the enhanced bioactivity of the prosclerotic cytokine TGF-β1 [33]. Therefore, sugar metabolism can influence TGF-β1 activity. Sugar metabolism is influenced by many factors, including enzymic regulation, induction, repression, and inactivation. This means that it is possible for other adaptations to alter the supply of nutrients [34]. We speculate that these genes influence the activity of the enzyme, which can lead to some differences in metabolism by influencing sugar metabolism.

## Conclusion

In this study, a total of 628 differently expressed genes were identified. Of these, 324 were up-regulated, and 304 were down-regulated in the P50 strain of *B*. *mori* compared to U11. We identified three pathways, including the proteasome, the Hippo signaling pathway, and amino sugar and nucleotide sugar metabolism, which showed significant differences between the two strains. This implies that the different expression of genes in these pathways leads to the difference in wing size between the two *B*. *mori* strains.

## Supporting information

S1 Table Primer sequences used for the qRT-PCR validation experiment. (XLSX)

S2 Table Summary of Illumina sequencing. (XLSX)

S3 Table DEGs between P50 and U11. (XLS)

S4 Table Annotated GO terms in wing disc transcriptome data. (XLS)

S5 Table Annotated KEGG pathways in wing disc transcriptome data. (XLS)

S6 Table *P*-value of qRT-PCR. (XLSX)
